# A case report of two instances of colorectal hepatoid adenocarcinoma, accompanied by a comprehensive literature review

**DOI:** 10.1007/s00432-023-05488-2

**Published:** 2023-11-10

**Authors:** Ran Zhang, Yi Yu, Jinxiu Zheng, Sijie Pi, Junhe Li, Jun Chen

**Affiliations:** 1 Department of Oncology, The First Affiliated Hospital, Nanchang University, Nanchang, 330006 Jiangxi China; 2 Department of Pathology and Pathophysiology, School of Basic Medical and Biological Sciences, Soochow University, Suzhou, 215000 Jiangsu China

**Keywords:** Colorectal cancer, Hepatoid adenocarcinoma, Alpha-fetoprotein, Pathology

## Abstract

**Objective:**

The study aimed to explore the clinical and pathological characteristics, survival outcomes, and prognostic factors of colorectal hepatoid adenocarcinoma.

**Methods:**

We performed two cases of colorectal hepatoid adenocarcinoma treated at the Oncology Department of the First Affiliated Hospital of Nanchang University. We also reviewed literature up to the present and performed a retrospective study of colorectal hepatoid adenocarcinoma.

**Results:**

Among the 39 patients included in this study, 28 had primary tumors in the colon, 9 in the rectum, and 2 in the rectosigmoid junction. The median age was 52 years (range 31–75 years); 28 patients (71.8%) were male. Out of the 32 patients for whom survival data were available, 24 patients succumbed to disease-related causes. The median overall survival of 32 patients was 8 months, with 1-year and 2-year overall survival rates of 31.0% and 16.0%, respectively. Univariate analysis revealed that depth of infiltration, presence of liver metastases, TNM stage, and the completeness of surgical resection were significantly associated with the overall survival period of colorectal hepatoid adenocarcinoma.

**Conclusion:**

Colorectal hepatoid adenocarcinoma exhibits a high degree of aggressiveness and poor prognosis. The major strategy for early-stage HAC was radical surgery and chemoradiotherapy demonstrates limited efficacy for extending survival.

**Supplementary Information:**

The online version contains supplementary material available at 10.1007/s00432-023-05488-2.

## Introduction

Hepatoid adenocarcinoma (HAC) is a rare histopathological subtype of adenocarcinoma characterized by prominent hepatocyte-directed differentiation and the characteristic secretion of alpha-fetoprotein (AFP). In 1970, Bourreille et al. (1970) reported the first case of gastric adenocarcinoma in a patient with an elevated serum AFP. The tumor showed a positive immunophenotype for AFP and histology presenting features of hepatocellular differentiation, known as an AFP-producing tumor. In 1985, Ishikura et al. (1985) first described gastric hepatoid adenocarcinoma, characterized by the production of AFP and the presence of a hepatoid differentiation pattern. In 1993, Nagai et al. (1993) clarified that the diagnosis of hepatoid adenocarcinoma should rely on the histological pattern, irrespective of elevated serum AFP and positive immunohistochemical AFP. Pathomorphologically, the tumor exhibits areas with typical adenocarcinoma features and hepatoid differentiation, characterized by a beam-like arrangement and the presence of large polygonal eosinophilic tumor cells in the hepatoid differentiation area (Kishimoto et al. 2000). Immunohistochemical staining can provide valuable information about the tumor cells’ response to specific markers, aiding in determining the origin and degree of differentiation of the tumor.

According to the previous literature (Ogbonna et al. 2016), the incidence of HAC ranged from 0.38 to 0.73%. HAC is most commonly found in the digestive system, particularly the stomach (83.9%), and less frequently in other sites, such as the gallbladder (3.7%), uterus (3.2%), lungs (2.3%), and bladder (1.8%). However, cases of colorectal hepatoid adenocarcinoma are extremely rare (< 0.5%) (Su et al. 2013). Treatment strategies for colorectal hepatoid adenocarcinoma typically involve a comprehensive approach including surgical resection, radiotherapy, and chemotherapy. However, due to the rarity and heterogeneity of the disease, there is currently a lack of standardized treatment options for colorectal hepatoid adenocarcinoma. The aggressive biological behavior of colorectal hepatoid adenocarcinoma contributes to an extremely poor prognosis, underscoring the importance of early diagnosis and prompt intervention.

We reported two cases of colorectal hepatoid adenocarcinoma treated at the First Affiliated Hospital of Nanchang University, and reviewed patients with colorectal hepatoid adenocarcinoma reported in the previous literature, with the objective of investigating the clinicopathological features, survival outcomes, and prognostic factors.

## Materials and methods

### Data sources and search strategy

We conducted a thorough literature search using the three major medical databases in China (CBM disc, HowNet, and Wanfang), as well as PubMed and Web of Science in English, covering the period from 1994 to December 2022. The following terms and their combinations were searched in the title/abstract field: (“Hepatoid” or “α-fetoprotein producing” or “alpha-fetoprotein producing”) AND (“cancer” or “carcinoma” or “adenocarcinoma”) AND (“colorectal” or “colon” or “rectum” or “intestinal”).

### Study selection criteria

The literature will be included if it meets the following criteria: (1) the diagnosis of colorectal HAC is confirmed by histopathological inspection; (2) there are clear therapies reported in articles (surgery or chemotherapy). The literature will be excluded if it meets the following criteria: (1) the case is not histopathologically diagnosed as primary colorectal HAC; (2) colorectal as the site of metastatic tumor; (3) lack of available full text in the literature. According to the inclusion and exclusion criteria, we selected 32 articles to read full texts, leading to the identification of 37 patients.

### Data extraction

Basic information of reports including name of the first author, year of publication, age of patients, and gender. Clinicopathological features, such as patient symptoms, tumor location and sizes, accompanying IBD, serum AFP levels, elevated of serum biomarkers, pathological data, TNM classification, treatment, and survival data, were collected.

### Statistical analysis

All statistical analyses were conducted with SPSS Statistics version 26.0 software (IBM, Armonk, NY, USA). Categorical variables were presented as numbers with percentages and continuous variables were presented as median with a range. The overall survival (OS) period was defined as the time from diagnosis to death (patients alive were censored at the last follow-up). Survival curves were calculated by the Kaplan–Meier (K–M) method, and the differences between survival curves were examined with log-rank test. Independent prognostic factors were analyzed by the Cox proportional hazard regression model. *P* < 0.05 as a threshold of statistical significance.

## Result

Our study presented two cases of colorectal hepatoid adenocarcinoma at the First Affiliated Hospital of Nanchang University, as well as conducted a retrospective analysis of 39 cases of colorectal hepatoid adenocarcinoma (including 37 cases reported since 1994 as identified by the search), to provide a comprehensive overview of the clinicopathological characteristics, survival outcomes, and prognostic factors associated with colorectal hepatoid adenocarcinoma.

### Case report

#### Case 1

**Fig. 1:**
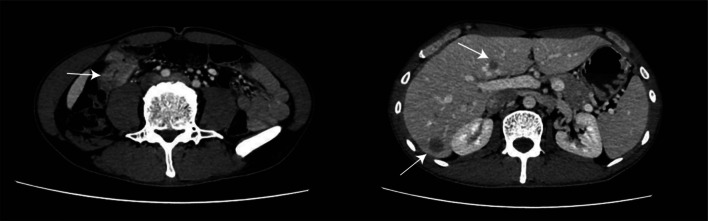
Representative CT-scan images of Case 1. In the hepatic area of the colorectal region, the presence of an occupation raises suspicion of colonic carcinoma. There were a few observed lymph-node metastases nearby, along with intrahepatic nodular shadows, suggesting the possibility of metastatic tumors

A 42-year-old male presented with increased frequency of bowel movements and underwent a CT-scan on November 2021, which revealed an occupation in the hepatic area of the colorectal region along with intrahepatic nodular shadows, prompting consideration of the possibility of colonic cancer with liver metastasis (Fig. 1).The patient had a family history of tumor, with no previous history of hepatitis. Enteroscopy performed at 60 cm from the anus indicated the likelihood of colonic carcinoma, biopsy pathology: moderately differentiated adenocarcinoma of the colon. Immunohistochemistry results indicated: pMMR; CerbB2 (1+); P53 (90% 3+); ki-67 (90%+). Laboratory tests, including liver and kidney function and blood routine, exhibited no significant abnormalities. However, alpha-fetoprotein levels were markedly elevated at 483.00 ng/ml, while CA199 was measured at 7.69 U/ml, CA125 at 7.68 U/ml, and CEA at 14.5 ng/ml. Genetic testing revealed wild-type K-ras, N-ras, and B-raf. The clinical staging was determined as T3NxM1 stage IV.

Subsequently, the patient received eight cycles of FOLFIRI in combination with bevacizumab [the last two cycles of treatment were temporarily halted following a multidisciplinary team (MDT) discussion regarding the feasibility of surgery], during which the efficacy was assessed to be PR on review, with a significant reduction in tumor size compared to the previous assessments. Additionally, the patient’s alpha-fetoprotein (AFP) levels continued to decline substantially, and the preoperative AFP was 37.9 ng/ ml.

**Fig. 2:**
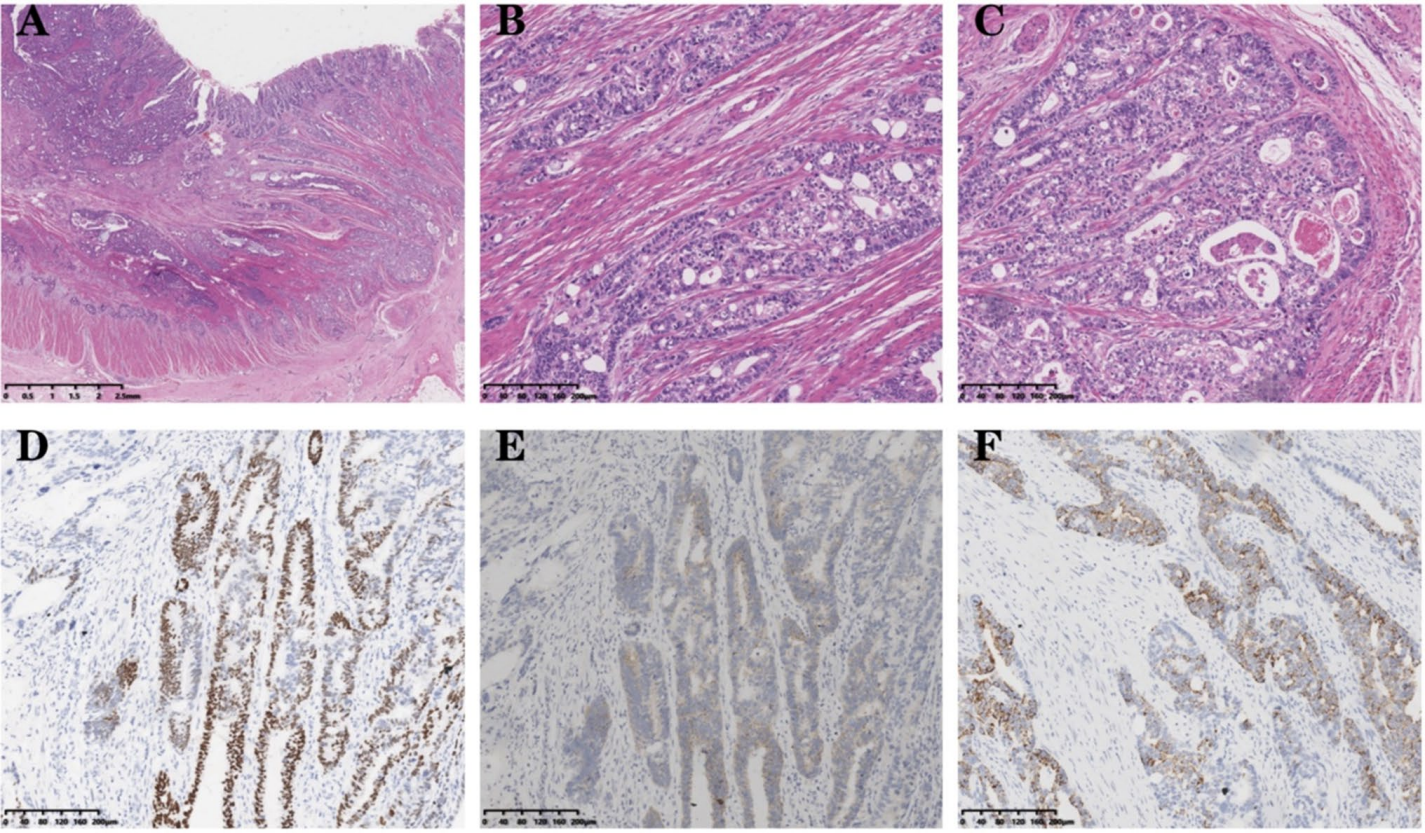
**A–C** Hematoxylin and eosin staining: the tumor tissue infiltrates the intestinal wall and displays pseudoadenoid structures. Plasma is present in the lumen, and endovascular tumor emboli are visible within the tumor. In the hepatic differentiation area, tumor cells are characterized by large polygonal shapes, eosinophilic cytoplasm, and vacuolated nuclei; **D** IHC:SALL-4 (Spalt-Like Transcription Factor 4), original magnification ×100; **E** IHC:GPC-3 (Glypican 3 proteoglycan), original magnification ×100. **F** IHC:Hep (Hepatocellular carcinoma-associated protein1), original magnification ×100

The patient underwent a right hemicolectomy 4 months after diagnosis. The specimen was then examined visually, revealing a bulging mass located 13 cm from the cut end of the ileum and 8 cm from the cut end of the colon. This mass occupied a significant portion of the intestinal canal, measuring 4 × 4 × 1 cm, and had a grayish-white and tough surface. Additionally, visible invasion of subplasma adipose tissues was observed. Tumor tissue was observed in the para-intestinal lymph nodes (11/39); additionally, six other cancerous nodules were identified. Microscopic view: The tumor predominantly consists of a combination of adenocarcinoma areas and hepatoid differentiation areas. Some regions of the adenocarcinoma areas exhibit a glandular tubular pattern with visible plasma and mucus in the tubular lumens. Focal necrosis is observed within the tumor, accompanied by abundant interstitial fibrous tissue, endovascular tumor emboli can also be identified. Hepatoid differentiated areas were organized in beams and nests. The tumor cells exhibited large polygonal shape with abundant eosinophilic cytoplasm. The nuclei were large and deeply stained, displaying noticeable heterogeneity. Additionally, some nuclei exhibited vacuolation (Fig. 2A–C). Immunohistochemistry showed SALL-4 (partial+), Hepatocyte (few weak+), and Gly-3 (partial+) (Fig. 2D–F), cerbB2 (2+), while FISH amplification was found to be negative. The diagnosis was as follows: (right hemicolon) Moderately-to-poorly differentiated adenocarcinoma with partial hepatoid adenocarcinoma differentiation. TNM staging: cT3N2bM1a, Stage IVA.

The patient refused further adjuvant therapy. Consequently, the serum alpha-fetoprotein (AFP) levels showed an upward trend, reaching 240 ng/ml, and CT scans indicated a significant increase in the size of intrahepatic metastatic lesions compared to previous assessments. Subsequently, the patient received three courses of FOLFOX combined with cetuximab. Upon reevaluation, the intrahepatic metastatic lesions were found to be stable compared to previous scans, and the AFP levels decreased to 150 ng/ml. Subsequently, the patient discontinued treatment due to financial constraints. The patient developed significant ascites, with CT revealed disease progression 6 months after the surgery. Then the patient succumbed to the disease, with an overall survival of 11.14 months.

#### Case 2

**Fig. 3:**
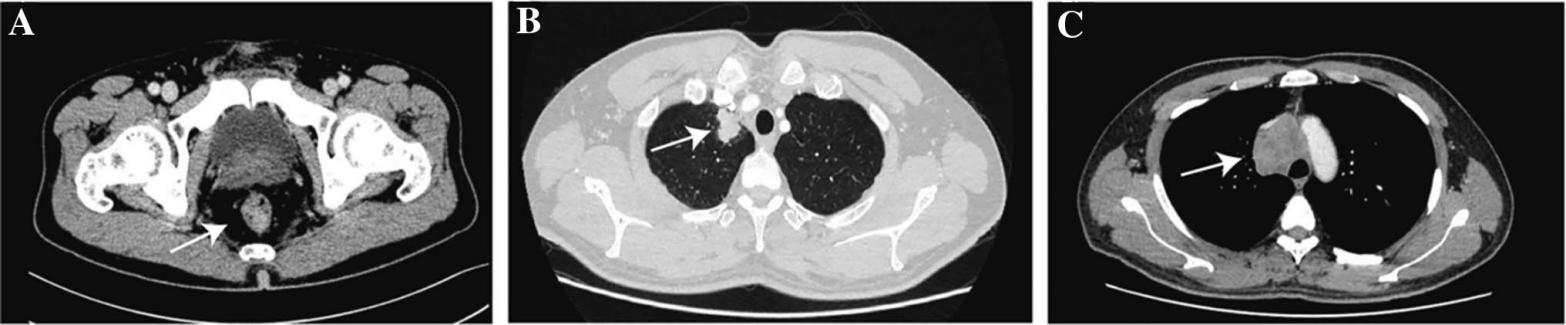
Representative CT-scan images of Case 2. **A** Occupation of the rectum, suggestive of rectal cancer, with invasion into the surrounding fat spaces, and no evidence of lymph-node or distant metastases observed. **B**, **C** The presence of an upper lobe right lung occupation along with enlarged mediastinal lymph nodes is indicative of malignancy and strongly suggests disease recurrence

A 51-year-old male presented with symptoms of bloody and dark stools. He had no prior history of hepatitis. The CT-scan conducted in November 2016 indicated rectal occupation, with a suspected diagnosis of rectal cancer. No signs of distant metastasis were observed on the CT-scan (Fig. 3A). Enteroscopy further revealed a substantial neoplasm located 5 cm from the anus, characterized by surface ulceration. This lesion occupied approximately half of the rectal cavity. Biopsy pathology confirmed a diagnosis of moderately differentiated carcinoma. Preoperative laboratory tests revealed no apparent abnormalities in liver and kidney function, complete blood count, or tumor markers (AFP: 2.95 ng/mL; CEA: 1.89 ng/mL; CA199: < 0.60 U/mL; CA125:7.87U/mL).

The patient subsequently underwent a laparoscopic transabdominal anterior resection + prophylactic terminal ileostomy for rectal cancer. Specimens are examined with the naked eye; a protruding mass was observed 2.1 cm from the cut end of the rectum, measuring 3.0 × 2.4 × 1.5 cm, with a gray-brown ring of tissue at the upper cut edge, measuring 2.3 × 1.7 × 1.1 cm. Immunohistochemistry results revealed the following: pMMR; CerB-2 (1+); EGFR (+). The diagnosis indicated grade II rectal adenocarcinoma, TNM staging: cT2N0M0, stage I (low grade with fewer than 12 lymph nodes). After the surgery, the patient received oral deoxyfluorouracil capsules for 6 months, followed by regular follow-up appointments, during which no significant recurrence or metastasis was detected.

On May 2022, the patient underwent a follow-up CT examination, revealing an occupied upper lobe of the right lung, suggestive of a malignant lesion. Enlarged mediastinal lymph nodes were also observed, raising concerns of metastasis (Fig. 3B,C). Additionally, Tumor marker AFP showed an elevation to 12.5 ng/ml. Subsequent aspiration biopsy of the lung occupancy and mediastinal lymph nodes confirmed the presence of adenocarcinoma, with rectal metastatic origin considered. Genetic testing indicated that KRAS/NRAS/BRAF were wild type. Based on these findings, tumor recurrence and metastasis were considered, leading to a revised diagnosis of stage IV.

**Fig. 4:**
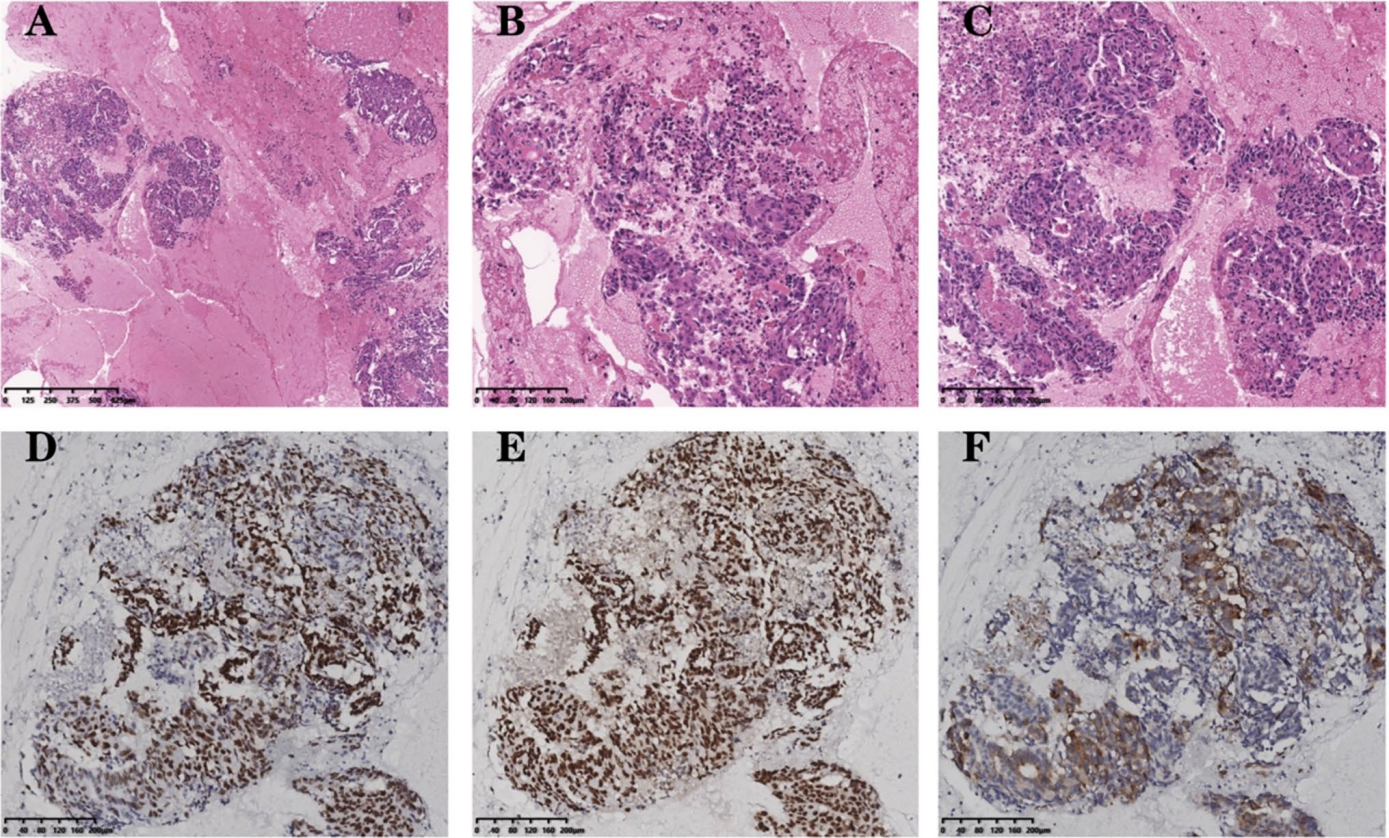
**A–C** Hematoxylin and eosin staining: the majority of tumor cells exhibited a large polygonal shape with abundant eosinophilic cytoplasm, large and deeply stained nuclei, evident heterogeneity, and presence of eosinophilic vesicles in the cytoplasm; IHC: SALL-4, original magnification ×100; IHC:BRG1 (Brahma-Related Gene 1), original magnification ×100; IHC:GPC-3, original magnification ×100

Then the patient was enrolled in “A Phase III clinical trial comparing the efficacy and safety of A140 and Epiduo in combination with the mFOLFOX6 regimen for the first-line treatment of patients with RAS wild-type metastatic colorectal cancer”. He received six cycles of the medication. Two months later, a follow-up CT-scan revealed a significant increase in lung foci and mediastinal multifocal fusion lymph nodes compared to the previous scan. Additionally, a new small node was observed in the upper lobe of the right lung, and the AFP levels persistently increased to 592 ng/ml. The treatment’s efficacy was assessed as progressive disease (PD), leading to the patient’s withdrawal from the clinical study. Considering the significantly elevated AFP levels in the patient, histopathological examination (HE) and immunohistochemistry were conducted. Microscopic evaluation revealed that the majority of tumor cells exhibited a large polygonal shape with abundant eosinophilic cytoplasm, large and deeply stained nuclei, evident heterogeneity, and the presence of eosinophilic vesicles in the cytoplasm (Fig. 4A–C). Immunohistochemistry analysis demonstrated partial positivity for Gly, and strong positivity for SALL4 and BRG1 (Fig. 4D–F). Hep was negative. These findings support the possibility of rectal hepatoid adenocarcinoma.

Subsequently, the patient underwent four cycles of FOLFIRI + Bevacizumab. During this period, there was a significant decrease in AFP levels, down to 303 ng/ml. Following these four cycles, the CT review revealed a significant enlargement of mediastinal lymph nodes and right lung nodules compared to previous scans. It also revealed superior vena cava involvement and the presence of new pericardial effusion. AFP levels had increased to 393 ng/ml from previous measurements. A pericardial effusion was obtained via puncture and showed positive chromosomal cytology, as well as the presence of malignant neoplastic cells. The treatment efficacy was assessed as progressive disease (PD). For financial reasons, the patient declined to undergo radiotherapy for the enlarged mediastinal lymph nodes. Instead, three courses of raltitrexed + fraquintinib were administered after experiencing disease progression, with concurrent pericardial perfusion chemotherapy. During this treatment, the patient’s AFP levels continued to decline, reaching 77 ng/mL. Subsequently, the patient discontinued chemotherapy due to deteriorating health. Finally, the patient succumbed to the disease progression 10 months after the recurrence.

### Retrospective study

Herein, we reported two cases of hepatoid adenocarcinoma which occurred in the colon and rectum respectively. Additionally, we collected the data of 37 cases with colorectal hepatoid adenocarcinoma reported in the literature (Wang et al. 2016, 2022; Fan et al. 2020; Zhang et al. 2013, 2005; Xu et al. 2019a, b, 2015; Zhou and Zhao 2019; Zhai et al. 2015; Ge et al. 2001; Xin et al. 2018; Hocking et al. 1995; Kurihara et al. 1997; Yachida et al. 2003; Fu et al. 2006; Anzai et al. 2015; Taguchi et al. 1997; Kato et al. 1996; Jun et al. 2016; Ishikura et al. 1997; Armaghani et al. 2015; Yuanyuan et al. 2014; Cappetta et al. 2012; Lattes et al. 2000; Levy et al. 2019; Slotta et al. 2012; Ming et al. 2018; Borgonovo et al. 2008; Liu et al. 2007, 2020; Sato et al. 1994). The clinicopathological characteristics of all 39 patients with colorectal hepatoid adenocarcinoma are summarized in Table 1.

**Table 1:** Clinicopathological characteristics of 39 colorectal hepatoid adenocarcinomas

Characteristic	*N* (%)
Sex (N = 39)	
Male	28 (71.8%)
Female	11 (28.2%)
Age, years (N = 39)	
≦ 60	25 (64.1%)
> 60	14 (35.9%)
History of other tumors (N = 39)	
Yes	3 (7.7%)
No	36 (92.3%)
History of ulcerative enteritis (N = 39)	
Yes	5 (12.8%)
No	34 (87.2%)
Symptoms (N = 39)	
Hematochezia	13 (33.3%)
Stomach pain	11 (28.2%)
Others [asymptomatic, change in bowel habit, positive fecal occult blood, etc.]	15 (38.5%)
Location (N = 39)	
Left hemicolon	10 (25.6%)
Right hemicolon	18 (46.2%)
Rectosigmoid junction	2 (5.1%)
Rectum	9 (23.1%)
Histological type	
Hepatoid adenocarcinoma	39/39 (100%)
Tumor size (N = 33)	
≦6 cm	22 (66.7%)
> 6 cm	11 (33.3%)
TNM stage (N = 39)	
I–II	6 (15.4%)
III	14 (35.9%)
IV	19 (48.7%)
Depth of violation (N = 29)	
T1–2	4 (13.8%)
T3	11 (37.9%)
T4	14 (48.3%)
Endovascular tumor emboli (N = 21)	
Yes	16 (76.2%)
No	5 (23.8%)
Lymph-node metastasis (N = 36)	
Yes	30 (83.3%)
No	6 (16.7%)
Distant metastasis (N = 39)	
No	20 (51.3%)
Yes	19 (48.7%)
Liver metastasis	17 (43.6%)
Surgical resection (N = 39)	
Complete resection	25 (64.1%)
Incomplete resection	13 (33.3%)
No surgery	1 (2.6%)
Elevated of serum biomarkers	
AFP (N = 32)	27 (84.4%)
CEA (N = 19)	11 (57.9%)
CA199 (N = 14)	6 (42.9%)
Serum AFP levels (N = 32)	
< 7 ng/ml	5 (15.6%)
7–500 ng/ml	5 (15.6%)
> 500 ng/ml	22 (68.8%)
Immunohistochemistry	
AFP for positive (N = 27)	24 (88.8%)
CEA for positive (N = 13)	11 (84.6%)
Hep for positive (N = 17)	11 (64.7%)
GPC-3 for positive (N = 12)	10 (83.3%)
KRAS/NRAS gene status (N = 9)	
Wild type	8 (88.9%)
Mutant type	1 (11.1%)

*TNM* tumor node metastasis, *AFP* alpha-fetoprotein, CEA carcinoembryonic antigen, *CA19-9* carbohydrate antigen 19-9, *Hep* Hepatocellular carcinoma-associated protein 1, *GPC-3* Glypican 3 proteoglycan, *KRAS* Kirsten rat sarcoma viral oncogene homolog, *NRAS* Neuroblastoma RAS viral oncogene homolog

### Patient clinicopathologic characteristics

Among the 39 patients diagnosed with colorectal hepatoid adenocarcinoma, 14 (14/39, 35.9%) were aged 60 or older, with a median age of 52 years (range 31–75 years). Of these patients, 28 (28/39, 71.8%) were male and 11 (11/39, 28.2%) were female (male-to-female ratio of approximately 2.55:1), and additionally, three patients (3/39, 7.7%) had a history of other neoplastic diseases, while five patients (5/39,12.8%) had a history of ulcerative enteritis. The most commonly reported symptom was blood in the stool (13/39, 33.3%), followed by abdominal pain (11/39, 28.2%). Other symptoms included changes in bowel habits, urgency, heaviness, and intestinal obstruction (15/39, 38.5%) (Table 1).

The pathological type of all 39 patients was colorectal hepatoid adenocarcinoma. 18 cases (46.2%) originated in the right hemicolon, 10 cases (25.6%) in the left hemicolon, 9 cases (23.1%) in the rectum, and 2 cases (5.1%) were located in the rectosigmoid junction. Tumor sizes were greater than 6 cm in 11 cases (11/33, 33.3%) and equal to or less than 6 cm in 22 cases (22/33, 66.7%). Of the 21 patients, 16 had endovascular tumor emboli (Table 1).

Out of the 39 patients, 6 (15.4%) were classified as stage I–II, 14 (35.9%) were classified as stage III, and 19 (48.7%) were classified as stage IV. In terms of tumor infiltration depth (T stage), there were 4 cases (4/29, 13.8%) in T1–2, 11 cases (11/29, 37.9%) in T3, and 14 cases (14/29, 48.3%) in T4. Regarding lymph-node metastasis (N stage), 30 cases (30/36, 83.3%) showed lymph node metastasis out of the 36 cases evaluated. Concerning distant metastasis (M staging), 19 patients (19/39, 48.7%) had distant metastasis, primarily in the liver, lung, peritoneum, and other sites. The liver was the most common site of metastasis, affecting 17 out of 39 patients (43.6%) (Table 1).

Among the 39 patients, 25 (25/39, 64.1%) underwent radical surgery, 13 (13/39, 33.3%)received palliative surgery, and 1 (1/39, 2.6%) did not undergo surgery (Table 1).

Among the 32 cases with reported alpha-fetoprotein levels, serum alpha-fetoprotein was found to be below 7 ng/ml in 5 cases (5/32, 15.6%), within the range of 7 ng/ml to 500 ng/ml in 5 cases (5/32, 15.6%), and exceeding 500 ng/ml in 22 cases (22/32, 68.8%). Serum CEA levels were elevated in 11 cases (11/19, 57.9%), while serum CA199 levels were elevated in 6 cases (6/14, 42.9%). Among the reported immunohistochemistry results, 24 cases (24/27, 88.8%) were AFP positive, 11 cases (11/13, 84.6%) were CEA positive, 11 cases (11/17, 64.7%) were Hep positive, 10 cases (10/12, 83.3%) were GPC-3 positive. Additionally, among the 9 patients who reported their genetic status, 8 (88.9%) had wild-type KRAS/NRAS, and 1 (11.1%) had a mutant.

### Survival analysis

**Fig. 5:**
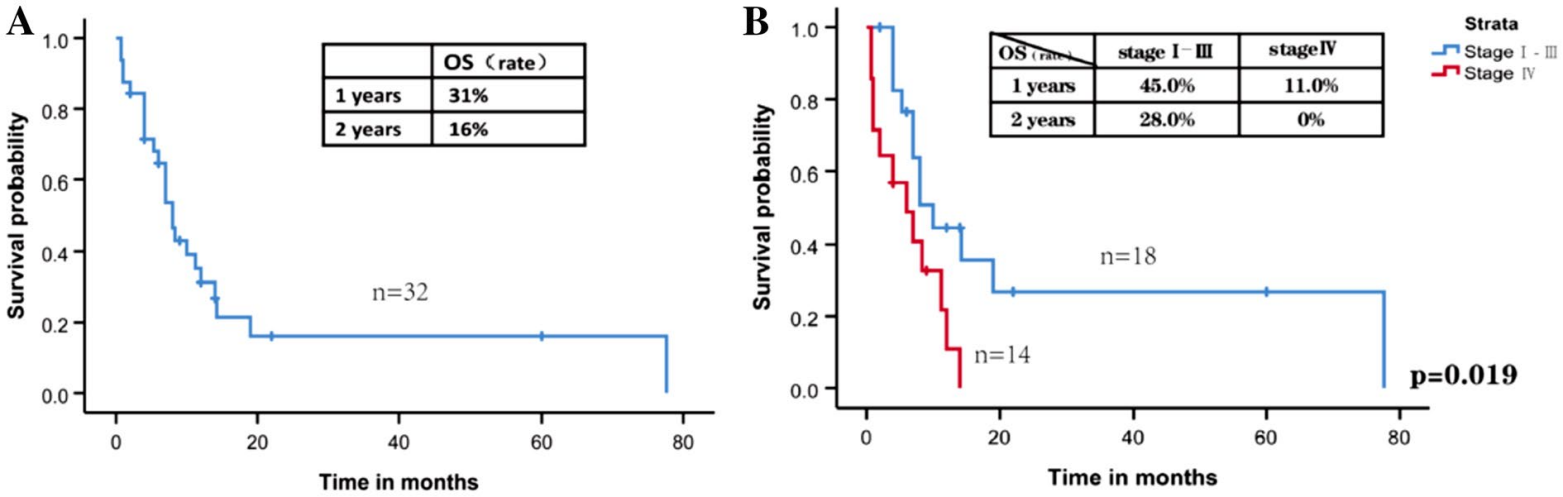
**A** Kaplan–Meier survival curve of overall survival of all patients. **B** Kaplan–Meier survival curve of overall survival of patients at stage I–III and stage IV

**Fig. 6:**
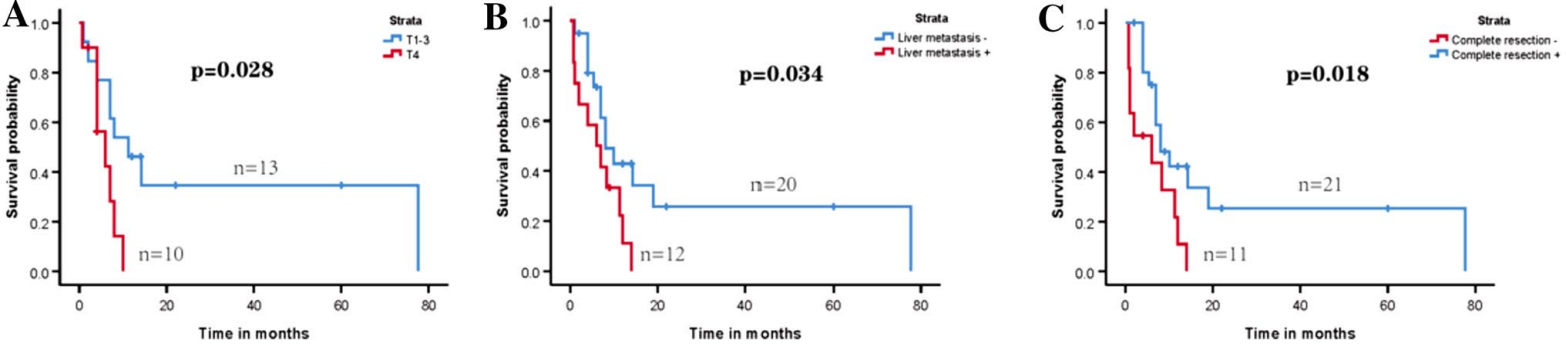
OS of patients of different subgroups. **A** OS of patients of varying degrees of invasion depth; **B** OS of patients with or without liver metastases; **C** OS of patients with or without complete resection

We conducted survival analysis according to the 32 patients (Table 2). The median duration of follow-up spanned 22 months (range from 0.7 to 77.6 months). Among them, 24 died due to illnesses. The median survival for these 32 patients was 8 months, with 1-year and 2-year overall survival rates of 31.0% and 16.0%, respectively (Fig. 5A). The 1-year and 2-year overall survival rates were 45.0% and 28.0% for patients in stages I-III, while patients in stage IV exhibited survival rates of 41.0%, 11.0%, and 0% at 6 months, 1 year, and 2 years (Fig. 5B). Further analysis indicated that patients with tumor of T1-3, without liver metastases, with complete resection tented to have longer overall survival (*P* = 0.028, *P* = 0.034, *P* = 0.018, respectively) (Fig. 6A–C).

**Table 2:** Survival data of the 32 cases with colorectal hepatoid adenocarcinoma

Survival characteristics	Parameter
Follow-up time (months)	
Mean (m ± SD)	36.4 ± 10.1
Median (m, range)	22.0 (0.7–77.6)
Survival data (N = 32)	
Disease-related deaths	24
Overall survival (N = 32)	
Mean (m ± SD)	18.9 ± 5.5
Median (m, range)	8.0 (0.7–77.6)
Survival rate (%)	
1-year overall survival rate	31.0%
2-year overall survival rate	16.0%
1-year/2-year overall survival for TNM stages 1–3	45.0/28.0%
6-month/1-year/2-year overall survival for TNM stage 4	41.0/11.0/0%

*SD* standard deviation

Table 3 displays the prognostic factors for overall survival. Univariate analysis identified depth of invasion (*P* = 0.045), liver metastases (*P* = 0.046), TNM stage (*P* = 0.028), and complete resection (*P* = 0.026) were significantly associated with poor survival outcome. However, multivariate analysis was not conducted due to the limited sample size.

**Table 3:** Prognostic factors for overall survival in patients with colorectal hepatoid adenocarcinoma based on univariate analysis

Prognostic factors	Univariate analysis	
	Hazard ratio (95% CI)	*P* value
Sex	1.253 (0.457–3.437)	0.661
Age	1.665 (0.725–3.824)	0.229
Location	1.075 (0.462–2.498)	0.867
Tumor size (N = 26)	1.590 (0.561–4.513)	0.383
Serum AFP levels (N = 27)	1.473 (0.491–4.415)	0.490
Depth of violation (N = 23)	3.065 (1.023–9.180)	0.045
Lymph-node metastasis (N = 30)	3.278 (0.754–14.259)	0.113
Liver metastasis	2.411 (1.016–5.720)	0.046
TNM stage	2.675 (1.115–6.420)	0.028
Complete resection	2.665 (1.124–6.316)	0.026

## Discussion

In our study, male accounted for a higher proportion of the entire group, with the ratio of male to female of approximately 2.55–1. The median age of the patients was 52 years. There was a greater prevalence of the disease in the colon compared to the rectum. The majority of the patients presented with symptoms, such as hematochezia and abdominal pain, which is consistent with previous reports. In the present study, we identified five patients with a combination of inflammatory bowel disease, (one in the colon, three in the rectum, and one in the rectosigmoid junction). Chronic inflammation has been proposed to have a role in this possible association (Levy et al. 2019). However, there is no literature reporting a specific mechanism linking inflammatory bowel disease to colorectal hepatoid adenocarcinoma.

Among the 32 patients who reported AFP levels in our study, 27 patients had elevated AFP levels. Serum AFP is a reliable marker for determining hepatocellular carcinoma and hepatoid adenocarcinoma. In most cases of colorectal hepatoid adenocarcinoma, elevated AFP levels are usually detected at very high levels (> 500 ng/ml), which also be accompanied by elevated serum CEA and CA199. Several studies (Inagawa et al. 2001; Wang et al. 2019) indicated that 70–80% of hepatoid adenocarcinoma (HAC) patients exhibit elevated serum AFP levels. The elevation was supposed to be associated with both the degree of tumor differentiation and, consequently, the prognosis. Furthermore, serum AFP levels have been recognized as a sensitive prognostic biomarker for both disease-free survival (DFS) and overall survival (OS). In our study, the AFP level of the patient in Case 1 was significantly elevated preoperatively, and decreased significantly postoperatively, then elevated again at the time of disease progression, which was consistent with the patient’s clinical regression. Therefore, some authors (Wang et al. 2022) suggest that, on the one hand, bowel cancer patients should undergo routine preoperative screening for AFP to assist in diagnosis. On the other hand, for patients with hepatoid adenocarcinoma accompanied by an elevated AFP, the AFP levels should be periodically reviewed to assist in determining tumor recurrence and metastasis.

While serum AFP levels are vital in hepatoid adenocarcinoma diagnosis, a conclusive diagnosis hinges upon a comprehensive evaluation that combines histological morphology and immunohistochemical markers. The preoperative diagnosis of hepatoid adenocarcinoma presents challenges due to the localization of hepatoid-differentiated cells deep within cancerous tissues, where they constitute a relatively small proportion of the tumor. Consequently, the detection rate of hepatoid-differentiated cancer cells through colonoscopic biopsy remains low (Zhang et al. 2013). Among the 39 patients in this study, 3 patients were conclusively diagnosed with colorectal hepatoid adenocarcinoma during the preoperative biopsy, 23 patients received clear diagnosis during the surgery, and 4 were definitively diagnosed by means of recurrent biopsy. The exact time of diagnosis was not reported for the remaining 9 cases. Hepatoid adenocarcinomas occurring in different sites share similar histomorphological characteristics. These tumors often consist of two types of lesions, with features of both hepatocellular carcinoma-like differentiation and adenocarcinoma differentiation. Migration between cells of the two components can be observed at the junction. The adenocarcinoma area typically exhibits well-differentiated tubular or papillary features. The morphology, arrangement, and organization of cells in the hepatocellular carcinoma-like differentiation zone closely resemble those observed in typical hepatocellular carcinoma. The cells exhibit a beam-like or solid sheet arrangement, characterized by large polygonal cells with eosinophilic cytoplasm. Additionally, within the cancer cells and the intercellular spaces, pale, eosinophilic, homogeneous, vitreous-like bodies may be observed (Liu et al. 2020). The nuclei of tumor cells in HAC appear vacuolated, often accompanied by prominent nucleoli (Wang et al. 2022).

In this study, endovascular tumor embolization was observed in 16 out of 21 patients, which is consistent with literature reports indicating that endovascular tumor embolization is present in 65–83% of hepatoid adenocarcinoma cases (Baek et al. 2011). This phenomenon is considered one of the factors contributing to the malignant biological behavior of hepatoid adenocarcinoma. However, the exact mechanism of action is not well understood. It is hypothesized that the cancerous cells produce alpha-fetoprotein (AFP), which up-regulates the expression of vascular endothelial growth factor (VEGF) proteins, leading to the formation of endovascular tumor embolization (Mukozu et al. 2013). Immunohistochemistry plays a crucial role in the diagnosis and differential diagnosis of hepatoid adenocarcinoma. The expression of hepatocellular carcinoma markers, including AFP, Arginase-1, Glypican-3 (GPC-3), SALL4, and Hep-1, provides valuable insights into the correlation with hepatocytes. AFP serves as a diagnostic aid, GPC-3 indicates hepatoid differentiation, while SALL4 is diffusely expressed in hepatoid adenocarcinoma but negative in hepatocellular carcinoma (Chandan et al. 2016; Ushiku et al. 2010), and additionally, Hep-1, another highly specific hepatocyte marker, demonstrates a sensitivity and specificity of 80% and 90%, respectively, making it a reliable indicator for hepatocyte differentiation and hepatocellular carcinoma diagnosis (Xu et al. 2019a). Therefore, the comprehensive analysis of histological patterns combined with the presence of different immunohistochemical markers is essential for accurate diagnosis and differential diagnosis of hepatoid adenocarcinoma.

The pathogenesis of Hepatoid Adenocarcinoma (HAC) remains uncertain. It primarily occurs in organs derived from the foregut derivatives of the primitive alimentary canal. Deviations in the differentiation process can lead to tumor differentiation toward hepatocytes (Ishikura et al. 1985). While the colorectum is derived from the midgut and hindgut during embryonic development, the presence of tubular adenocarcinoma and areas of hepatoid differentiation may be attributed to the adenoepithelial and hepatoid differentiation potential of stem cells in the colorectum (Zhang et al. 2013).

Moreover, it is necessary to differentiate colorectal hepatoid adenocarcinoma from the following tumors: (1) Metastatic hepatocellular carcinoma: Hepatoid adenocarcinoma exhibits similarities in histomorphology, immunophenotype, and serum AFP levels to hepatocellular carcinoma (HCC), making differentiation challenging. However, in general, HCC originates from chronic liver disease with a history of hepatitis or cirrhosis, and it lacks the typical components of intestinal adenocarcinoma. Furthermore, immunohistochemically, hepatoid adenocarcinoma shows positive expression of SALL4, whereas HCC exhibits negative expression. Therefore, SALL4 can serve as a specific and effective marker for differentiating hepatoid adenocarcinoma from HCC (Ushiku et al. 2010). (2) Hepatocellular yolk cystic tumor: the tumor cells exhibit hepatoid differentiation, characterized by enlarged size, abundant cytoplasm with eosinophilia, and the presence of visible hyaline globules. The nuclei are centrally located, and the degree of anisotropy can vary, with eosinophilic nucleoli being observed. Unlike hepatoid adenocarcinoma, which predominantly occurs in children or young individuals, particularly young women, a diligent search for the classic morphology of yolk cystic tumor reveals papillomatous, ballooning structures, or a reticular and lax structure (Liu et al. 2020). (3) Fetal gastrointestinal adenocarcinoma: Fetal gastrointestinal adenocarcinoma is characterized by tumor morphology that closely resembles the fetal intestine at 12 weeks of gestational age. It exhibits distinct subnuclear vacuoles, positive expression of embryonic intestinal mRNA and AFP, and negative expression of Heppar1. The tumor is characterized by a morphology that lacks hepatocyte-like differentiation and presents as tubular structures with prominent subnuclear vacuoles (Liu et al. 2020). The differentiation still requires a comprehensive evaluation by considering clinical history, imaging studies, endoscopic examination, and immunohistochemical analysis results.

Colorectal hepatoid adenocarcinoma has a higher incidence of lymph-node and distant metastasis, particularly liver metastasis, which is often detected at an advanced stage. In this study, out of the 39 patients, 19 were diagnosed with stage IV disease at the time of initial diagnosis. Previous studies have shown that AFP-positive hepatoid adenocarcinoma is more prone to liver metastasis, and higher AFP levels are associated with a higher rate of liver metastasis (Lin et al. 2015). In our study, the patient in case 1 presented with markedly elevated serum AFP levels and liver metastasis upon initial diagnosis. The present study also demonstrated a significant association between liver metastasis (*P* = 0.046) and poor prognosis. The prognosis of patients with hepatoid adenocarcinoma, regardless of AFP elevation, is generally worse than that of ordinary adenocarcinoma. The study suggested that approximately two-thirds of patients died within 1 year, with a median survival of 8 months. Stage IV patients (19/39) showed an extremely poor prognosis, with most patients succumbing within 6 months and a 1-year survival rate of only 11.0%. These findings are consistent with the previous reports in the literature.

Due to the extreme rarity of this disease, the current treatment approach for colorectal hepatoid adenocarcinoma is similar to that for conventional intestinal adenocarcinoma, including surgical resection of the primary tumor and oligometastatic lesions, chemoradiotherapy, as well as palliative care. Radical surgery is considered the preferred treatment option for prolonging patient survival. In this study, it was observed that patients who underwent complete resection had significantly better outcomes compared to those who did not (*P* = 0.018), and that patients who received early radical surgery achieved longer survival times. Chemotherapy regimens frequently incorporate combinations of FOLFOX (leucovorin, 5-fluorouracil, and oxaliplatin) or FOLFIRI (leucovorin, 5-fluorouracil, irinotecan), along with the antiangiogenic agent bevacizumab. The epidermal growth factor receptor cetuximab is also conducted in patients with KRAS/NRAS wild-type tumors, with varying outcomes. For example, Cappetta et al. (2012) reported a 2-month progression-free survival for a 75-year-old female patient using FOLFIRI plus bevacizumab after relapse. And Jun et al. (2016) described a 66-year-old male patient who had a poor response to standard treatment (FOLFOX-4 and later FOLFIRI plus bevacizumab), showing significant disease progression and an increase in serum AFP from 149.8 to 7049.1 ng/ml. In contrast, Yuanyuan et al. (2014) reported a 36-year-old male patient with disease relapse who experienced a significant decrease in serum AFP after receiving FOLFOX plus bevacizumab, and 6 months later, achieving stable lesion control. In our study, the patient in Case 1 seemed sensitive to FOLFIRI combined with bevacizumab treatment, resulting in a substantial decrease in serum AFP and significant tumor shrinkage. The patient in Case 2 also exhibited comparable response to the standard treatment regimen. Some studies (Petrelli et al. 2012; Sun et al. 2022) have explored alternative treatment options for hepatoid adenocarcinoma due to its similarities with hepatocellular carcinoma. However, due to the rarity and aggressiveness of this tumor, further research is needed to establish evidence-based therapeutic approaches.

## Conclusion

Colorectal hepatoid adenocarcinoma is a rare disease that can be easily misdiagnosed or underdiagnosed in clinical practice. Its pathogenesis is still unclear, but its highly malignant biological behavior, poor treatment response, and unfavorable prognosis should be given attention. Early diagnosis and surgical treatment are crucial for improving the survival of patients with colorectal hepatoid adenocarcinoma. It is important to note that the sample size in this study is relatively small, and long-term follow-up data are limited, which may not fully represent all the biological behaviors of this type of tumor in the intestinal tract. Therefore, more cases need to be accumulated for further analysis. Nevertheless, we hope that this study can enhance clinicians’ understanding of this particular type of tumor and promote the use of radical surgical resection, ultimately improving patient prognosis.

## Limitations

First, the sample size of the data in this study was insufficient, which may introduce statistical bias. Second, due to the retrospective nature of the study, the range of patient data collected was limited.

## Supplementary Information

Below is the link to the electronic supplementary material.

Supplementary file1 (DOC 75 KB)

## Data Availability

All data generated or analyzed during this study are included in this published article.
