# Supplementary material for: A case report of two instances of colorectal hepatoid adenocarcinoma, accompanied by a comprehensive literature review
Source: J Cancer Res Clin Oncol. 2023 Nov 10;149(20):18241–52. doi: 10.1007/s00432-023-05488-2 (PMC10725335; doi:10.1007/s00432-023-05488-2)
Supplement: Supplementary file 1 — Supplementary file1 (DOC 75 KB) [file 432_2023_5488_MOESM1_ESM.doc]

**Appendix A**

Table A1. All selected articles according to our inclusion and exclusion criteria.

| Case | First author | Year | Gender | Age |
| --- | --- | --- | --- | --- |
| 1 | WANG Han | 2022 | 1 | 44 |
| 2 | LIU You | 2020 | 1 | 31 |
| 3 | XU Hui-rong | 2019 | 0 | 51 |
| 4 | ZHOU Jian-xun | 2019 | 0 | 65 |
| 5 | XU Zi-wei | 2019 | 1 | 54 |
| 6 | ZHAI Xiao-li | 2019 | 1 | 73 |
| 7 |  |  | 1 | 70 |
| 8 |  |  | 1 | 68 |
| 9 | ZHANG Ji-xin | 2013 | 1 | 37 |
| 10 | ZHANG Jie | 2005 | 1 | 50 |
| 11 | XU Yuan-yuan | 2015 | 1 | 52 |
| 12 | YOSHlHlRO SATO | 1994 | 1 | 43 |
| 13 | WANG Zhi-hong | 2016 | 1 | 74 |
| 14 |  |  | 1 | 51 |
| 15 | Hiroyuki Anzai | 2015 | 0 | 41 |
| 16 | Avan Armaghani | 2015 | 0 | 42 |
| 17 | Zhi Xin | 2018 | 1 | 61 |
| 18 | GE Wei-lu | 2000 | 0 | 53 |
| 19 | JUN TAGUCHI | 1997 | 1 | 71 |
| 20 | Kazuya Kato | 1996 | 1 | 75 |
| 21 | Kuangi Fu | 2006 | 1 | 71 |
| 22 | Alessandro Cappetta | 2011 | 0 | 75 |
| 23 | Yuanyuan Chen | 2014 | 1 | 36 |
| 24 | Ming Hu | 2018 | 1 | 63 |
| 25 | Giacomo Borgonovo | 2008 | 1 | 42 |
| 26 | C. Lattes | 2000 | 1 | 41 |
| 27 | LOU Ke-xin | 2020 | 0 | 60 |
| 28 | Shinichi Yachida | 2003 | 1 | 59 |
| 29 | Jan Erik Slotta | 2012 | 0 | 59 |
| 30 | Alexander N. Levy | 2019 | 1 | 38 |
| 31 | Q Liu | 2007 | 0 | 50 |
| 32 | H.ISHIKURA | 1997 | 0 | 48 |
| 33 | Hocking, Glenn R | 1995 | 0 | 39 |
| 34 | KATSUMI KURIHARA | 1997 | 1 | 67 |
| 35 | JUN LI | 2016 | 1 | 66 |
| 36 |  |  | 1 | 49 |
| 37 |  |  | 1 | 62 |
| 38 | OUR CASE |  | 1 | 42 |
| 39 |  |  | 1 | 51 |

Included case reports. Cases are reported in progressive number. Legend: 1 = male, 0 = female. Age is expressed in years.
